# Accumulation of oncometabolite D-2-Hydroxyglutarate by SLC25A1 inhibition: A metabolic strategy for induction of HR-ness and radiosensitivity

**DOI:** 10.1038/s41419-022-05098-9

**Published:** 2022-07-22

**Authors:** Kexu Xiang, Christian Kalthoff, Corinna Münch, Verena Jendrossek, Johann Matschke

**Affiliations:** Institute of Cell Biology (Cancer Research), University Hospital Essen, University of Duisburg-Essen, 45147 Essen, Germany

**Keywords:** Radiotherapy, Cancer metabolism, Non-small-cell lung cancer, CNS cancer, Targeted therapies

## Abstract

Oncogenic mutations in metabolic genes and associated oncometabolite accumulation support cancer progression but can also restrict cellular functions needed to cope with DNA damage. For example, gain-of-function mutations in isocitrate dehydrogenase (IDH) and the resulting accumulation of the oncometabolite D-2-hydroxyglutarate (D-2-HG) enhanced the sensitivity of cancer cells to inhibition of poly(ADP-ribose)-polymerase (PARP)1 and radiotherapy (RT). In our hand, inhibition of the mitochondrial citrate transport protein (SLC25A1) enhanced radiosensitivity of cancer cells and this was associated with increased levels of D-2-HG and a delayed repair of radiation-induced DNA damage. Here we aimed to explore the suggested contribution of D-2-HG-accumulation to disturbance of DNA repair, presumably homologous recombination (HR) repair, and enhanced radiosensitivity of cancer cells with impaired SLC25A1 function. Genetic and pharmacologic inhibition of SLC25A1 (SLC25A1i) increased D-2-HG-levels and sensitized lung cancer and glioblastoma cells to the cytotoxic action of ionizing radiation (IR). SLC25A1i-mediated radiosensitization was abrogated in MEFs with a HR-defect. D-2-HG-accumulation was associated with increased DNA damage and delayed resolution of IR-induced γH2AX and Rad51 foci. Combining SLC25A1i with PARP- or the catalytic subunit of DNA-dependent protein kinase (DNA-PKcs)-inhibitors further potentiated IR-induced DNA damage, delayed DNA repair kinetics resulting in radiosensitization of cancer cells. Importantly, proof of concept experiments revealed that combining SLC25A1i with IR without and with PARPi also reduced tumor growth in the chorioallantoic membrane (CAM) model in vivo. Thereby SLC25A1i offers an innovative strategy for metabolic induction of context-dependent lethality approaches in combination with RT and clinically relevant inhibitors of complementary DNA repair pathways.

## Introduction

Cancer cells frequently harbor defects in DNA repair proteins, including core proteins of DNA double-strand break (DSB) repair by non-homologous end joining (NHEJ) or homologous recombination (HR) repair [1–3]. Such cancer-cell-specific alterations in core DSB repair proteins provoke vulnerability to drugs interfering with respective alternative DSB repair pathways and thus offer opportunities for efficient cancer cell killing by a process called “synthetic lethality”. The concept of synthetic lethality has been developed for tumors harboring loss of function mutations in genes coding for two proteins of HR repair, *BRCA1* or *BRCA2* [4–7]: *BRCA1/2*-mutant tumors were characterized by defects in HR-repair (“BRCAness” or “HR-ness”) and this defect turned out to be synthetically lethal with inhibition of poly (ADP-ribose) polymerase (PARP)-dependent DNA repair pathways using PARP-inhibitors. The effects of synthetically lethal compounds in cancer cells with DSB repair defects may be further enhanced by the addition of DNA damage-inducing treatments, e.g., radiotherapy (RT) or genotoxic chemotherapy [8]. This may be important since BRCA1/2 mutant cancer cells can also develop resistance against PARP-inhibitors highlighting the need for novel therapeutic approaches to prevent or overcome resistance [9]. In addition, the success of such approaches requires the identification and validation of robust molecular markers predicting response or resistance to inhibitors of the DNA damage response and DNA repair.

During recent years, the identification of cancer-associated mutations in genes with impact on cellular metabolism has attracted considerable attention [10]. Some of these mutations (e.g., isocitrate dehydrogenase (IDH), Succinate dehydrogenase (SDH), fumarate hydratase (FH)) lead to the abnormal production of metabolites associated with cancer progression, called “oncometabolites” [11]. Aberrant production of the oncometabolites 2-hydroxyglutarate (2-HG), succinate, and fumarate in cancer was linked to cellular metabolic transformation and disturbance of biological processes [7, 12, 13]. As an example, 2-HG accumulation has been observed in glioblastoma patients harboring gain-of-function mutations in IDH [14], resulting in aggressive phenotype and increased susceptibility to radiotherapy (RT) treatment [15]. Mechanistically, the biological effects of 2-HG accumulation have been linked to inhibition of alpha-ketoglutarate (α-KG) dependent dioxygenases (α-KGDD) [11]. Among others, these enzymes include JmjC domain-containing histone demethylases (KDMs) that disturb histone demethylation and repair of ionizing radiation (IR)-induced DNA damage [16]. Specifically, in cells with somatic gain-of-function mutation of IDH, 2-HG accumulation resulted in inhibition of a histone-lysin demethylase KDM4B and associated disturbance of HR repair [16], thus sensitizing cancer cells to PARP inhibition with clinical relevance [7].

In the above scenario, 2-HG accumulation and associated HR-ness occurred in the context of a genetic defect of cancer cells [7].

Our previous work revealed a contribution of mitochondrial citrate carrier SLC25A1 to acquired radioresistance of cancer cells with tolerance to chronic cycling hypoxia [6]. Moreover, analysis of publicly available datasets revealed that lung cancer patients with high SLC25A1 expression had a poorer prognosis [6]. Pharmacological inhibition of SLC25A1 by 1,2,3-benzene-tricarboxylic acid (BTA) treatment enhanced radiosensitivity of lung cancer cells and delayed repair of radiation-induced DNA damage [6].

Interestingly, pharmacological inhibition of SLC25A1 increased cellular D-2-hydroxyglutarate (D-2-HG) levels. We, therefore, hypothesized that SLC25A1 inhibition may decrease functionality of HR repair as a result of D-2-HG accumulation. Thus, SLC25A1i may provide a yet unknown opportunity for metabolic induction of a HR-ness phenotype and resulting in context-dependent lethality with DNA repair inhibitors of end joining (EJ) dependent DNA repair pathways and potentially also in combination with RT. Here, we selected lung and glioblastoma cell lines without described mutation in IDH to investigate if treatment with a more specific 3rd generation small molecule SLC25A1 inhibitor CTPI2 [17] would induce D-2-HG accumulation and mimic the HR-ness phenotype of IDH1/2 deficient cells. Moreover, we analyzed if treatment with the SLC25A1 inhibitor would result in metabolically induced HR-ness phenotype and context-dependent lethality in combination with DNA damage induction by ionizing radiation (IR) in combination with inhibitors of end joining DNA repair pathways, e.g., inhibitors of PARP or of the catalytic subunit of DNA dependent protein kinase (DNA-PKcs). Our data demonstrated the potential of CTPI2 treatment to increase radiosensitivity with DNA repair inhibitors of EJ pathways in vitro and in vivo through induced accumulation of the oncometabolite D-2-HG.

## Materials and methods

### Cell culture and reagents

Human NSCLC cell lines A549 and NCI-H460, human glioblastoma cell lines U87-MG and T98G were cultured in DMEM (+D-Glucose, +L-Glutamine, -Pyruvate, Invitrogen) media supplemented with 10% FBS and 1% Penicillin-Streptomycin (Sigma-Aldrich) at the condition of humidified incubator at 37 °C and 5% CO_2_. These four cell lines were obtained from ATCC (Bethesda, MD, USA), STR typed, and tested for mycoplasma regularly. In addition, we used the isogenic pairs of mouse embryonic fibroblasts (MEF) without or with deficiency in a critical proteins of NHEJ (Lig IV^−/−^) or HR repair pathways (Rad54^−/−^), respectively [18]. These cells were provided by Dr. Frederick W. Alt as a gift.

### Quantification of D-2-HG

D-2-Hydroxyglutarate (D-2-HG) Assay Kit (Colorimetric) (BioVision, Milpitas, CA, USA) was applied to quantify the intracellular D-2-HG levels according to the manufacturer’s protocol. In brief, 10 million cells were homogenized, lysed, and spun down. Supernatant was collected and transferred to 96-well plate followed by measuring the enzymatic conversion of D-2-HG to α-KG which could interact with the probe and produce a detectable colored product. Absorbance at 450 nm was measured with a BioTek Synergy H1 Microplate reader (BioTek Instruments, Inc., Winooski, VT, USA).

### Transfection and treatment

To achieve downregulation of SLC25A1 on protein level, cells were transfected with 45 nM siRNA pools targeting SLC25A1 or non-targeting controls (SMARTpool: ON- TARGETplus, Dharmacon/Horizon Discovery, Cambridge, UK) with 3 μl TransIT-siQUEST (Mirus Bio, Madison, WI, USA) and 100 μl optiMEM (Gibco/Life Technologies, Carlsbad, CA, USA) for 24 h in 1 ml total reaction volume, followed by medium exchange according to manufacturer’s protocols as previously described [19]. Twenty-four after start of transfection experiments were performed. Treatment with drugs was performed 2 h before irradiation by using the following concentrations: (i) CTPI2 (200 µM), (ii) PJ34 (4 µM), NU7447 (4 µM).

### Protein quantification by western blot

Anti-rabbit SLC25A1 Polyclonal antibody (#PA5-42451, Thermo Fisher, Rockford, IL, USA) and anti-mouse β-actin from Sigma Aldrich (St. Louis, MO, USA, #A1978) were used for Western blot analysis. After harvesting, cells were lysed in 75 µl of RIPA buffer containing 0.5% Sodiumdesoxycholate, 1% NP-40 (Nonidet), 0,1% Sodiumdodecylsulfate (SDS), 50 mM Tris-HCl, 150 mM NaCl, 5 µg/ml aprotinin, 5 µg/ml leupeptin, and 3 µg/ml pepstatin as described previously [6]. Protein was separated by 12% sodium dodecyl sulfate polyacrylamide gel electrophoresis (SDS-PAGE) under reducing conditions and transferred onto polyvinylidenefluoride (PVDF) membranes (Roth, Karlsruhe, Germany). Blots were blocked in RotiBlock (Roth, Karlsruhe, Germany) for 1 h at room temperature. The membranes were incubated overnight at 4 °C with the respective primary antibodies. The secondary antibody was incubated for 1 h at room temperature. Detection of antibody binding was performed by enhanced chemiluminescence (ECL Western Blotting Analysis System; GE Healthcare/Amersham Biosciences, Freiburg, Germany). Densitometry analysis was performed using ImageJ 2.00 (National Institutes of Health, Bethesda, MD, USA).

### Assessment of DNA damage

A549, NCI-H460, U87-MG, T98G cell lines were plated in 12 well plates with the density of 200.000 cells per well. Treatment was administrated 24 h after plating at different concentrations. Slides were covered with 1% low melting point (LMP) agarose to form the first layer for gel retention. The second or cell-containing layer was a mixture of 30% cell containing media and 70% 1% LMP agarose. Slides were placed in a lysis solution (Content:1.2 M NaCl, 100 mM Na_2_EDTA, 0.1% sodium lauryl sarcosinate and 0.26 M NaOH, pH > 13) for 1 h at 4 °C after the agarose gel became solid. Then the slides were put into freshly prepared alkaline electrophoresis solution (Content: 2 mM Na_2_EDTA and 0.03 M NaOH, pH = 12.3) for 10 min before electrophoresis. Electrophoresis was performed for 1 h at 20 V. After that, slides were immersed in water and 100% ethanol to drain excess electrophoresis solution. Propidium iodide (PI) was used to detect the DNA under fluorescence microscopy.

### γH2AX/Rad51 foci detection

Cells were fixed with fix/permeabilization solution (4% PFA and 0.2% Triton X-100 in PBS) for 15 min at room temperature after treatment and then blocked by block solution (2% normal goat serum (NGS) in PBS) for 30 min at room temperature. For γH2AX staining, cells were stained by γH2AX antibody (Alexa Fluor® 647 Mouse anti-H2AX (pS139), BD Bioscinces, USA, #AB_1645414), which coupled to Alexa Flour 647, at 1:50 dilution in block solution for 1 h at room temperature. For Rad51 staining, 1:100 Rad51 rabbit (Anti-Rad51 (Ab-1) Rabbit pAb, Millipore, USA, #PC130) in blocking solution was utilized to incubate for 30 min on a shaker at 4 °C. After that, anti-rabbit secondary antibody (Goat anti-Rabbit IgG (H + L) Highly Cross-Adsorbed Secondary Antibody, Alexa Fluor Plus 488, Thermo Fisher, USA, # A32731) with the dilution of 1:400 in blocking solution was used for staining for 75 min at room temperature. Nuclear foci were evaluated manually under the AxioObserver.Z1 Fluorescence Microscope (with Apotome) (Zeiss, Oberkochen, Germany).

### Quantification of long-term survival

We performed standard colony formation assays to assess the long-term survival upon treatment as previously described [20, 21]. Different cell densities (200, 400, 800, 1600, and 3200 cells per well) were plated in 6-well plates and treated with indicated drug concentrations 24 h later. Radiation was performed 2 h after drug treatment with doses of 2, 5, and 8 Gy separately. Non-irradiated cells were sham irradiated at room temperature for the same period of time as their irradiated counterparts. 8–10 days later, colonies were stained with methanol containing 0.1% (w/v) Coomassie Blue dye and counted manually.

### Determination of KDM4 activity

30.000 cells were plated in 6 well plates 24 h before treatment. Drug treatment was performed for 24 h and nuclear extracts of treated cells were prepared according to the instructions of the manufacturer (ab113474 – Nuclear Extraction Kit, abcam, Cambridge, UK). The, KDM4 activity was determined in nuclear extracts by a fluorescence-based method according to the manufacturer’s instructions (ab113462 –KDM4/JMJD2 Activity Quantification Kit, abcam, Cambridge, UK).

### Analysis of cell function by flow cytometry

#### Determination of cytoplasmic and mitochondrial reactive oxygen species (ROS), apoptosis and cell death

30.000 cells were plated in six well plates 24 h before treatment. The supernatant of cells was collected in flow cytometry tubes before detaching the cells with accutase (PAN Biotech, Germany). Accutase reaction was stopped by the addition of cell culture media. Afterwards, detached cells were aliquoted into 3 flow cytometry tubes and pelleted at room temperature at 1500 rpm. The supernatant was discarded and cell-pellets were stained with following staining solutions separately for different purpose: (a) Cytoplasmic ROS levels: 0.5 µM dihydroethidium (DHE, Invitrogen, USA) diluted in PBS; (b) Mitochondrial ROS levels: 5 µM MitoSOX-Staining Solution (Invitrogen, USA) diluted in DMEM (+D-Glucose, +L-Glutamine, −Pyruvate) media; (c) Apoptosis levels: 5 µg/ml PI diluted in hypotonic buffer (0.05% Triton X-100 + 0.1% Natriumcitrat in PBS); (d) Cell death levels: 1 µg/ml PI diluted in PBS. CytoFLEX Flow Cytometer (Beckman Coulter, Inc. U.S.A) and FACS Calibur (Becton Dickinson, Heidelberg, Germany) were employed to evaluate samples.

#### Determination of DNA damage by γH2A.X foci analysis

For quantification of DNA damage, we recorded the time-dependent formation and resolution of γH2AX foci using flow cytometry, the cells were fixed with Fix-Perm-Solution (Bioscience™ Foxp3/Transcription Factor Staining Buffer Set, Invitrogen, USA) for 1 h before stained with γH2AX staining solution for 0.5 h. γH2AX staining solution was consist of γH2AX antibody (γH2AX (Alexa Fluor 647), BD Pharmingen, USA, #AB_1645414) at the ratio of 1:100 in permeable buffer (Bioscience™ Foxp3/Transcription Factor Staining Buffer Set, Invitrogen, USA). After staining, cells were transferred into flow cytometry tubes for measurement. Finally, γH2AX score was calculated as follows:

$$\gamma {\rm{H2AX}}\,{{{\rm{score}}}} = \% {\gamma} {\rm{H2AX}}\,{{{\rm{positive}}}}\,{{{\rm{cells}}}}^\ast {{{\rm{fluorescence}}}}\,{{{\rm{intensity}}}}\,{{{\rm{of}}}}\,{{{{\gamma}}{\rm{H2AX}}}}\,{{{\rm{positive}}}}\,{{{\rm{cells}}}}$$

Here, the γ-H2AX foci readouts obtained by flow cytometry were confirmed by the γ-H2AX foci readouts collected by the fluorescence microscopy method as described before [6].

### Determination of living cell numbers

We used a standard crystal violet assay to quantify the amount of living cells, indicative of cell viability and proliferation as previously described [6, 19, 22]. Therefore, we seeded 5.000 cells per well in a 96 well plate and incubated cells for 24 h at 37 °C before treatment. Media were discarded and cells were fixed after the respective treatment period using 1% glutaraldehyde. After fixation, cells were stained with 0.1% crystal violet staining solution and lysed by the addition of 0.2% Triton-X 100 in PBS. Absorbance at 540 nm was measured with BioTek Synergy H1 Microplate reader (BioTek Instruments, Inc., Winooski, VT, USA).

### Cell redox state determination

NAD^+^, NADP^+^, NADH, and NADPH levels, as well as NAD+/NADH and NADP^+^/NADPH ratios were examined using the NAD/NADH-Glo™ or NADP/NADPH-Glo™ Assays kit (Promega, USA) according to the manufacturer’s protocols. Briefly, 10.000 cells were plated in a 96 well plate 24 h before treatment. Cells were lysed followed by measuring NAD(P)^+^ or NAD(P)H luminescence signal separately by using BioTek Synergy H1 plate reader (Biotek, USA). The ratio of NAD+/NADH and NADP+/NADPH were calculated based on the instruction of the kits.

### Assessment of mitochondrial function (Seahorse technology)

We seeded 10.000–15.000 cells in each well, except the four corners for background correction, of a seahorse XF 96 well plate and incubated at 37 °C in 5% CO_2_ overnight. The cell culture medium was replaced with 180 μl of Seahorse XF DMEM Media containing 5 mM HEPES and supplemented with 1 mM Pyruvate, 2 mM Glutamine, 10 mM Glucose. After media exchange, cells were incubated at 37 °C in a CO_2_ free incubator for 45 min before measurement. Oxygen consumption rates (OCR) and extracellular acidification rate (ECAR) were measured by Seahorse XF96 Analyzer (Agilent, Santa Clara, USA). OCR was determined at four consecutive steps: [1] without any treatment, after injection of [2] oligomycin (1 μM), [3] carbonyl cyanide 4-(trifluoromethoxy) phenylhydrazone (FCCP, 2 μM), [4] Rotenone and Antimycin A (0.5 μM). After each assay, cells were stained with Hoechst (200 µM) for 5 min and fluorescence was measured by using BioTek Synergy H1 plate reader (Biotek, USA). Hoechst read out was used for normalizing the measured OCR/ECAR values to cell numbers as previously described [21].

### Determination of tumor growth in vivo

We used the Chick Embryo Chorioallantoic Membrane (CAM) to investigate the effects of treatment on tumor growth in vivo. Tumor grafting on the CAM was conducted as previously described [23–25]: In brief, Chicken eggs were incubated in the environment of relative air humidity of 65% and a temperature of 37 °C with a static position for 10 days before grafting. On the grafting day, large vessel area was marked before opening the “window”. A hole was created at the bottom of the eggs with scissor, widen with tweezer. The chosen “window” was opened by using a drill. For the grafting, 2 × 10^6^ cells were resolved in 50 μl PBS and grafted onto the CAM. Tumors were dissected and its diameters were measured 7 days after grafting.

### Statistical evaluation

Statistical analysis was generated using GraphPad Prism 7.0, calculations of various formulas mostly using Microsoft Excel 2019. Experiments were at least repeated three times and groups were defined by comparable variances. Assuming a normal distribution, statistical significances were calculated. For this, either the unpaired Students t-test or the two-way ANOVA (analysis of variance, means comparison test) with a post hoc test according to Bonferroni was used. The confidence interval was set to 95%. The significance level was set at *α* = 0.05 (equivalent to 5%), i.e., the difference between two data sets is significant if the *p* value ≤ 0.05. Significances were marked with asterisks (*) in the Figures. Here, *p < 0.05 stands for significant, **p < 0.01 for highly significant, ***p < 0.001 for extremely significant and ****p < 0.0001 for most significant, ns for non-significant.

## Results

### Inhibition of SLC25A1 leads to accumulation of D-2-HG and potentiates DNA damage upon exposure to IR

**Fig. 1:**
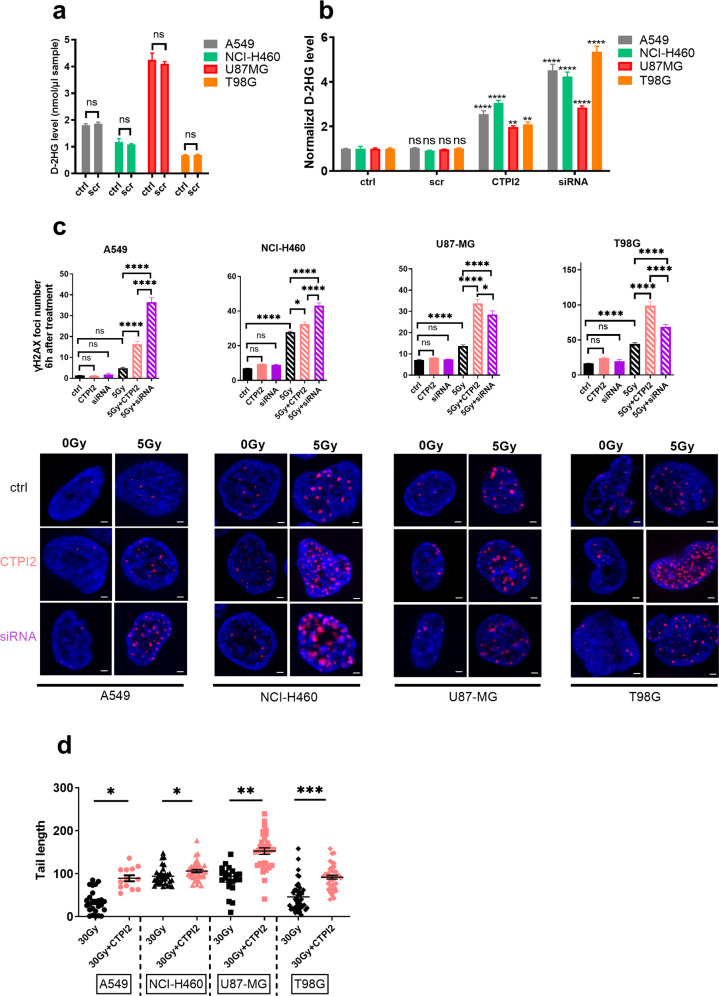
Genetic and pharmacologic SLC25A1 inhibition induced D-2-HG accumulation and enhanced radiation-induced DNA damage. A549, U87-MG, T98G, and NCI-H460 cells were pre-treated for 2 h with CTPI2 (200 μM) or siRNA alone and irradiated with an indicated radiation dose or left untreated (ctrl). To achieve downregulation of SLC25A1 on protein level, cells were transfected with 45 nm siRNA pools targeting SLC25A1 or non-targeting scrambled controls (scr) with 3 μl TransIT-siQUEST and 100 μl optiMEM for 24 h in 1 ml total reaction volume, followed by medium exchange according to manufacturer’s protocols. **a** Basal D-2-HG production in A549, NCI-H460, U87-MG, and T98G cell lines was investigated by using the D-2-HG assay kit. **b** Levels of D-2-HG production in A549, NCI-H460, U87-MG, and T98G cell lines were determined by using the D-2-HG assay kit 6 h after CTPI2 treatment or 24 h after RNAi-mediated SLC25A1 downregulation. Bars depict mean values of three independent experiments normalized to basal levels of the respective cell line. **c** *Upper panels:* γ-H2AX foci number was counted 6 h after combinatory treatment with IR (5 Gy) without or with prior SLC25A1 inhibition by CTPI2 or RNAi in A549, NCI-H460, U87-MG, and T98G cell lines. *Lower panels:* Representative pictures of nuclear γ-H2AX in A549, NCI-H460, U87-MG, and T98G cell lines as indicated. Blue, Hoechst 33342; red, γ-H2AX. Scale bar: 2 μm. **d** DNA damage induced by SLC25A1 inhibition (CTPI2) with IR (30 Gy) in A549, NCI-H460, U87-MG, and T98G cell lines was determined by the alkaline comet assay 6 h after CTPI2 treatment. Data represent the mean values (±SD) from three independent experiments (*N* = 3). Statistical significance: by non-parametric unpaired t-test. ns = not significant (*p* > 0.05), *p < 0.05, **p < 0.01, ***p < 0.001, ****p < 0.0001.

First, we investigated if we could reproduce our earlier findings on the link between SLC25A1 inhibition and D-2-HG accumulation in other cancer cells. Therefore, we downregulated the expression of SLC25A1 by siRNA-technology (RNAi) (Fig. S1a) and determined the effects on D-2-HG levels in lung (A549, NCI-H460) and glioblastoma (U87-MG, T98G) cell lines as described previously [19]. Though the cell lines displayed various basal D-2-HG-levels (Fig. 1a), genetic inhibition of SLC25A1 by RNAi significantly enhanced D-2-HG production in all four cell lines (Fig. 1b). The U87-MG cell line was characterized by the lowest induction of D-2-HG when calculated as fold-change compared to other cell lines, presumably due to the high basal D-2-HG level (Fig. 1a). Next, we tested the effects of a novel 3^rd^ generation small molecule inhibitor of SLC25A1 (CTPI2) on D-2-HG induction [17, 26]. Pharmacologic inhibition of SLC25A1 by CTPI2 for 6 h significantly stimulated D-2-HG levels compared to non-treated control cells, thus reproducing the stimulatory effects of the genetic downregulation of SLC25A1 by siRNA (Fig. 1b). However, pharmacologic inhibition was less potent in stimulating D-2-HG accumulation compared to genetic inhibition by RNAi, particularly in T98G cells with low basal D-2-HG levels (Fig. 1b). Time-resolved analysis of D-2-HG modulation in NCI-H460 cells further revealed that the increase in D-2-HG levels was detectable for up to 48 h, though highest D-2-HG accumulation was observed 6 h after CTPI2 treatment. These data corroborate our earlier findings that pharmacologic inhibition of SLC25A1 induces a pronounced and sustained D-2-HG accumulation (Fig. S1b) [6].

Next, we aimed to determine if SLC25A1-inhibition by RNAi or CTPI2 would affect radiation-induced DNA damage and repair as suggested by our earlier investigations. Therefore, we first determined the impact of both treatments on the induction and resolution of radiation-induced DNA damage by quantifying γ-H2AX foci as a marker for DNA DSBs [27, 28]. Genetic (SLC25A1-RNAi) or pharmacologic (CTPI2) SLC25A1 inhibition indeed enhanced the number of radiation-induced γ-H2AX foci in all tested cell lines, when determined 6 h after IR (Fig. 1c). This effect was particularly high in A549 cells with low basal radiosensitivity and low in NCI-H460 cells with high basal radiosensitivity. Interestingly, in the two lung cancer cell lines the effects of genetic inhibition on the levels γ-H2AX were more pronounced compared to CTPI2 treatment, thus paralleling the differences in the effects of the two treatments on the D-2-HG accumulation (Fig. 1b, c). Instead, the effects of CTPI2 treatment were rather similar to SLC25A1-RNAi in the U87MG cells, again reflecting the comparable effects of both treatments on D-2-HG accumulation. In T98G cells, the numbers of γ-H2AX foci at 6 h after irradiation reached the highest levels among the investigated cell lines (Fig. 1c). However, treatment of cell lines with CTPI2 or SLC25A1-RNAi induced no effects on the formation of γ-H2AX foci without IR (Fig. 1c). Importantly, SLC25A1 inhibition not only enhanced initial levels of radiation-induced DNA damage, at least in A549, U87-MG, and T98G cells, but also retarded the resolution of γ-H2AX foci in all tested cell lines with cell-line and treatment specific kinetics (Fig. S1c). This resulted in higher numbers of residual γ-H2AX foci 24 h after IR (Fig. S1c).

To verify the suggested increase in residual DSBs, we additionally performed alkaline comet assays [29] in lung cancer and glioblastoma cells exposed to irradiation with a single high dose (30 Gy) without or with CTPI2 pre-treatment. CTPI2-treatment significantly enhanced radiation-induced DNA damage compared to irradiation alone in all cell lines investigated (Fig. 1d). Again, we observed cell line specific variations on DNA damage levels 6 h after IR alone or in combination with pharmacologic SLC25A1 inhibition, corroborating the data obtained with γ-H2AX foci analysis. Importantly, time-resolved analysis of the effects of CTPI2 treatment on radiation-induced DNA damage revealed that SLC25A1 inhibition augmented the levels of radiation-induced DNA damage in NCI-H460 cells as of 0.5 h after irradiation reaching maximum levels at 4 h and (Fig. S1d); again, DNA damage levels did not reach basal levels 24 h after irradiation suggesting sustained effects of metabolic inhibition on the repair of radiation-induced DNA damage.

### Radiosensitzing effects of the SLC25A1 inhibitor CTPI2 are only observed in HR proficient but not in HR deficient MEF

**Fig. 2:**
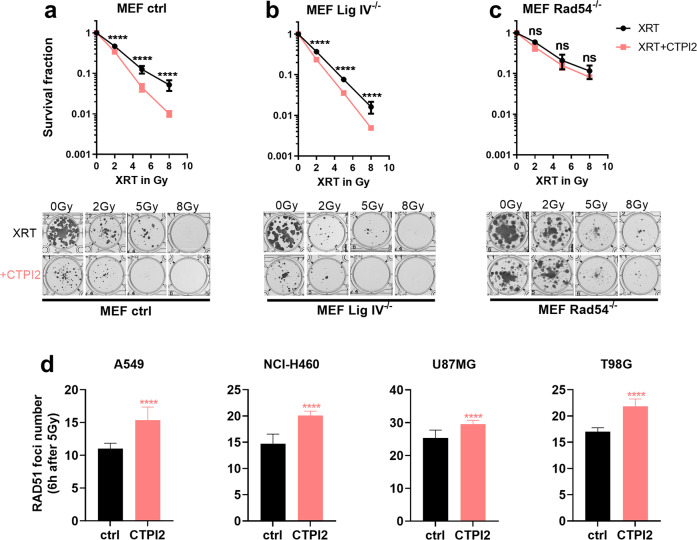
CTPI2 radiosensitized HR proficient but not HR deficient MEFs and enhanced accumulation of Rad51 foci in irradiated cancer cells. Wild-type and mutant MEF cell lines were pre-treated for 2 h with CTPI2 (200 μM) or left without drug treatment and then irradiated with a dose of 2 Gy, 5 Gy, 8 Gy or left unirradiated as indicated. **a**–**c** *Upper panels:* Survival fraction determined by standard colony formation assays was calculated 8 days after respective treatment in MEF wild-type cell line (**a**), MEF Lig IV^−/−^ (defect in NHEJ) cell line (**b**) and in MEF Rad54^−/−^(defect in HR) cell line (**c**). *Lower panels:* representative pictures of colony formation after irradiation as indicated. **d** Rad51 foci number was counted 6 h after IR treatment (5 Gy) alone or in combinatory treatment of IR (5 Gy) and CTPI2 in A549, NCI-H460, U87-MG, and T98G cell lines. Data represent the mean values (±SD) from three independent experiments (*N* = 3). Statistical significance: Two Way ANOVA with a post hoc test according to Bonferroni, and non-parametric unpaired t-test were applied here. ns = not significant (*p* > 0.05), ***p < 0.001, ****p < 0.0001.

So far, our data demonstrated that genetic and pharmacologic SLC25A1 inhibition induces an accumulation of the oncometabolite D-2-HG and impair the repair of radiation-induced DNA damage. The strong effect of CTPI2 treatment on the slower phase of DSB repair and prior work from others suggested that D-2-HG accumulation would disturb HR repair, potentially on the level of KDM4B-mediated histone 3 lysine 9 trimethylation (H3K9me3) near DNA breaks [16]. To challenge our assumption, we investigated potential differences in the effects of pharmacologic SLC25A1 inhibition on radiosensitivity of HR proficient and HR deficient cells. Therefore, we used isogenic MEF deficient in NHEJ due to deficiency in the critical NHEJ enzyme Ligase IV (Lig IV^−/−^ MEFs) or in HR repair caused by a deficiency in the HR protein Rad54 (Rad54^−/−^ MEFs) and the respective control MEFs without DNA repair defect [18] and monitored long-term survival in combination with SLC25A1 inhibition by CTPI2 by standard colony formation assays (Fig. 2a–c). Interestingly, treatment with CTPI2 significantly sensitized MEF cells with intact HR repair pathway (MEF ctrl, Fig. 2a, MEF Lig IV^−/−^, Fig. 2b), but did not alter the survival of irradiated Rad54^−^^/−^ MEF cells (Fig. 2c). The observation that CTPI2 sensitized control MEFs and NHEJ-deficient MEFs but failed to sensitize HR deficient MEFs suggested that CTPI2 most likely acted in the same pathway like Rad54-deficiency, thus mimicking a HR-ness phenotype.

To verify our assumption on CTPI2-mediated suppression of HR repair, we additionally quantified Rad51 foci as a marker for DNA repair by HR in A549, NCI-H460, U87-MG, and T98G cells exposed to irradiation with 5 Gy (Figs. 2d; S1e). SLC25A1 inhibition significantly enhanced the number of Rad51 foci when analyzed at 6 h after irradiation and also retarded the resolution of Rad51 foci (Figs. 2d; S1e). The CTPI2-induced impairment of Rad51 foci resolution in irradiated cancer cells corroborate a disturbance of HR repair by SLC25A1 inhibition.

Finally, we explored if CTPI2 treatment and associated D-2-HG accumulation was able to reproduce the KDM4 inhibition observed downstream of 2-HG accumulation in IDH-deficient cancer cells [16]. As demonstrated by KDM4/JMJD2 Activity Quantification Kit, CTPI2-treatment significantly decreased the activity of KDM4 enzymes as determined in NCI-H460 cells, supporting a role of KDM4-inhibition and associated effects on DNA repair in the effects of SLC25A1 inhibition (Fig. S1f).

### Treatment with the oncometabolite D-2-HG phenocopies the effects induced by CTPI2 on DNA damage, DNA repair, and cell survival upon irradiation

**Fig. 3:**
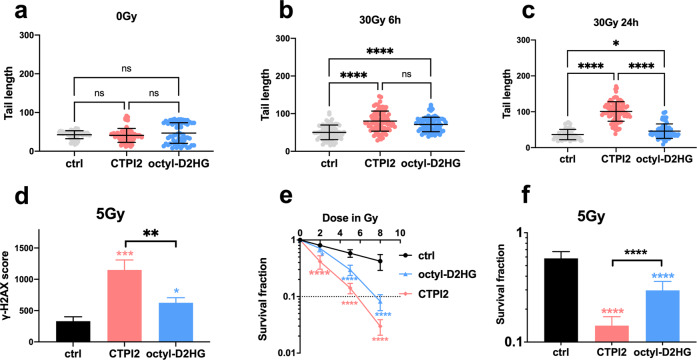
Octyl-D-2-HG-treatment enhanced radiation-induced DNA damage and radiosensitized NCI-H460 cell lines. NCI-H460 cells were pre-treated for 2 h with solvent, CTPI2 (200 μM) or octyl-D-2-HG (150 μM) and subsequently exposed to irradiation with 0–30 Gy as indicated. DNA damage was quantified by alkaline comet assay **a**, **c** 24 h or **b** 6 h after the indicated treatments. **d** γ-H2AX signal was assessed by flow cytometry 6 h after the indicated treatment. **e** Colony formation assay was used to verify the survival fraction of NCI-H460 cells upon combined treatment as indicated. **f** Survival fraction (SF) of control (ctrl), CTPI2, or octyl-D-2-HG treatment groups in combination with IR (5 Gy) determined by colony formation assay. Data represent the mean values (±SD) from three independent experiments (*N* = 3). Statistical significance: one way ANOVA followed by Bonferroni post-test. ns = not significant (*p* > 0.05), *p < 0.05, ***p < 0.001, ****p < 0.0001.

The above findings indicated that SLC25A1 inhibition amplified radiation-induced DNA-damage. To gain more mechanistic insight into the CTPI2-induced effects on DNA repair and radiosensitivity, and the role of D-2-HG accumulation herein, we explored if we could reproduce some of the CTPI2 effects by treating NCI-H460 cells with octyl-D-2-HG, a cell membrane-permeable form of D-2-HG. While single treatment of NCI-H460 cell line with octyl-D-2-HG or CTPI2 did not induce DNA damage as determined by the alkaline comet assay (Fig. 3a), combining octyl-D-2-HG with IR amplified radiation-induced DNA damage at 6 and 24 h after exposure to IR (Fig. 3b, c). However, residual DNA damage induced by CTPI2 treatment was significantly higher compared to membrane-permeable D-2-HG treatment (Fig. 3c).

Next, we compared the ability of cells to repair radiation-indued DSBs upon CTPI2 or octyl-D-2-HG treatment by quantifying radiation-induced γ-H2AX foci using flow cytometry [28]. Again, treatment with CTPI2 or octyl-D-2-HG in combination with irradiation with a single dose of 5 Gy led to an increase in the number of γ-H2AX compared to irradiation alone. However, in line with the data obtained by the alkaline Comet assay (Fig. 3b), the effects of octyl-D-2-HG treatment on the number of γH2AX foci determined 6 h after irradiation with a single dose of 5 Gy, which was lower compared to CTPI2-treatment (Figs. 3d, S2m). Nevertheless, CTPI2- and octyl-D-2-HG-treatment maintained irradiation-induced DNA damage state at a higher level, supporting an inhibitory effect of both treatments on DNA repair.

Investigating the long-term effect of CTPI2- and octyl-D-2-HG- treatment on the radiosensitivity of NCI-H460 cells treatment with both, CTPI2 and octyl-D-2-HG, significantly decreased the survival fraction (SF) of NCI-H460 cells in combination with IR-treatment (Fig. 3e). Though CTPI2-treatment was again more effective in reducing the SF of irradiated NCI-H460 cells compared to octyl-D-2-HG treatment (Fig. 3e, f), both treatments increased radiation-induced DNA damage and thus radiosensitized NCI-H460 cells.

### Octyl-D-2-HG or CTPI2 treatment disrupts mitochondrial metabolism and cellular function

**Fig. 4:**
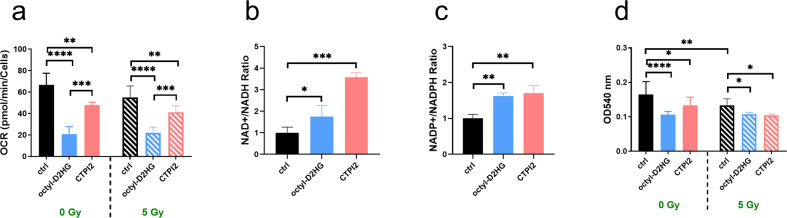
SLC25A1 inhibition by CTPI2-treatment or octyl-D-2-HG altered cell metabolism, viability, and function in NCI-H460 cell line. NCI-H460 cells were treated with CTPI2 (200 μM), octyl-D-2-HG (150 μM), or solvent control with 0 Gy or with single irradiation with 5 Gy. **a** Basal respiration of mitochondrial function was measured 24 h after treatment with or without IR using the mitochondrial stress test and the Seahorse XF96 Extracellular Flux analyzer. NAD and NADP kits were utilized to determine the ratio of NAD+/NADH (**b**), NADP+/NADPH (**c**) in NCI-H460 cells 24 h after treatment. **d** Crystal violet assay was applied to determine cell proliferation and viability 24 h after treatment. Data represent the mean values (±SD) from three independent experiments (*N* = 3). Statistical significance: one way ANOVA followed by Bonferroni post-test. ns = not significant (*p* > 0.05), *p < 0.05, **p < 0.01, ***p < 0.001, ****p < 0.0001.

Previous work demonstrated an involvement of SLC25A1 in mitochondrial energy metabolism with potential impact on the repair of radiation-induced DNA damage [6]. Therefore, we also compared the impact of SLC25A1-inhibition and associated D-2-HG accumulation to the effects induced by octyl-D-2-HG supplementation on mitochondrial function using Seahorse XF96 Extracellular Flux analyzer. The utilization of mitochondrial stress test revealed that both CTPI2- and octyl-D-2-HG-treatment for 24 h inhibited basal (Figs. 4a, S2n), maximal respiration (Fig. S2a, n), and mitochondrial ATP production (Fig. S2b, n), irrespective of additional IR-treatment.

Since SLC25A1 is involved in redox and energy homeostasis [30], the ratio of NAD^+^/NADH and NADP^+^/NADPH was monitored. CTPI2-treatment increased the ratio of NAD^+^/NADH and NADP^+^/NADPH, indicating a more oxidative state of NCI-H460 cells (Fig. 4b, c). Similar results were obtained by octyl-D-2-HG treatment (Fig. 4b, c). Furthermore, investigating the relative amount of monitored co-Enzymes and energy carrier-molecules NAD and NADP revealed a reduction of the amount of these metabolites either upon CTPI2 or octyl-D-2-HG treatment (Fig. S2c, d).

Finally, we determined the effect of both treatments on cell proliferation and viability by using the crystal violet assay as previously described [6, 19]. In agreement with functional mitochondrial impairment, proliferation of NCI-H460 cells was reduced 24 h after CTPI2- or octyl-D-2-HG-treatment (Fig. 4d).

In order to gain deeper insight into potential differences between CTPI2 or octyl-2-HG treatment on short-term cell function and survival we employed flow cytometry to compare treatment-induced effects on cytosolic and mitochondrial ROS as well as on apoptosis and cell death levels. CTPI2-treatment enhanced the number of cells with cytoplasmic (Fig S2e, i) and mitochondrial ROS production (Fig. S2f, j) compared to non-treated control NCI-H460 cells with or without IR-treatment. In line with deregulated co-Enzymes and energy carrier-molecules NAD and NADP, these data point to a general induction of cellular oxidative stress by CTPI2- or octyl-D-2-HG-treatment. Furthermore, the CTPI2- or octyl-D-2-HG-treated NCI-H460 cancer cells experienced enhanced apoptosis (Fig. S2g, k) and cell death levels (Fig. S1h, l) without and in combination with additional IR treatment. Thus, the effects observed upon CTPI2 or octyl-D-2-HG-treatment on short-term cell function seemed to be rather similar, although CTPI2-treatment was more effective on DNA repair and radiosensitivity.

Taken together, CTPI2- or octyl-D-2-HG-treatment lowered mitochondrial function and shifted the balance of the co-enzymes and energy carrier-molecules NAD and NADP to an oxidative state compared to untreated controls. This pronounced metabolic reprograming may contribute to the effects on cell proliferation and short-term survival of NCI-H460 cells described above.

### Pharmacologic inhibition of SLC25A1 by CTPI2 sensitizes cancer cells to treatment with inhibitors of end joining pathways alone or in combination with IR in vitro and in vivo

So far, our data indicated that SLC25A1 inhibition by CTPI2 induced a global metabolic reprogramming with accumulation of D-2-HG (Fig. 1). Accumulation of D-2-HG was associated with reduced ability to remove radiation-induced DNA DSB, presumably by inhibiting KDM4 activity and thereby disturbing HR repair (Fig. 2). We, therefore, speculated that the suspected metabolic induction of a HR-ness phenotype should sensitize cancer cells to combined treatment with inhibitors of complementary end joining dependent DNA repair pathways, e.g., the PARP1-dependent alternative end joining (alt-EJ), or the DNA-PKcs-dependent NHEJ. Thus, we investigated if combining CTPI2 treatment with the PARP-inhibitor (PARPi) PJ34 or the DNA-PKcs inhibitor (DNA-PKi) NU7447 would sensitize irradiated lung (A549, NCI-H460) and glioblastoma (U87-MG and T98G) cancer cell lines to radiation treatment.

**Fig. 5:**
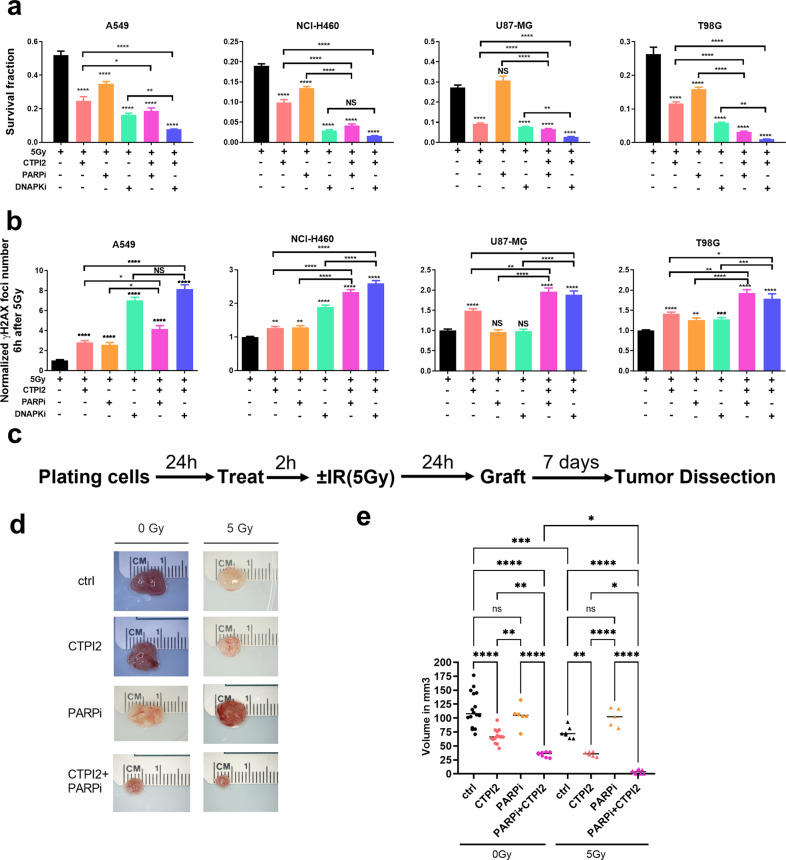
SLC25A1 inhibition combined with end joining (EJ) inhibitors potentiates DNA damage, reduces long-term survival and in vivo tumor growth in combination with IR. A549, U87-MG, T98G, and NCI-H460 cells were pre-treated for 2 h with CTPI2 (200 μM), PARPi (PJ34, 4 µM), DNAPKi (NU7447, 4 µM) or their combinations as indicated, and irradiated with a dose of 5 Gy. **a** Survival of clonogenic tumor cells was determined by standard colony formation assays 8 days after treatment. Data depict surviving fractions. **b** Numbers of γ-H2AX foci were counted in the 4 cell lines 6 h after combinatory treatment as indicated. Data depict γ-H2AX foci numbers normalized to 0.5 h time point. **c** Treatment scheme for the chicken chorioallantoic membrane (CAM) assay: NCI-H460 cells were plated in flasks 2 h before CTPI2 (200 μM), PARPi (PJ34 at 4 μM), or CTPI2 + PARPi treatment. Cells were irradiated with a dose of 5 Gy 2 h after drug-treatment or stayed without IR. After 24 h, cells were detached from the flask and grafted onto the CAM of the eggs. Tumors were dissected 7 days after grafting. **d** Exemplary photomicrographs of NCI-H460 tumors dissected from CAM model 7 days after grafting representing the indicated treatments. **e** Quantification of tumor volumes obtained in the respective treatment groups. Data represent the mean values (±SD) from three independent experiments (*N* = 3). Statistical significance: by non-parametric unpaired t-test. *p < 0.05, **p < 0.01, ***p < 0.001, ****p < 0.0001.

CTPI2 treatment increased eradication of clonogenic A549, NCI-H460, U87-MG, and T98G cells upon irradiation. Similarly, DNA-PKi alone resulted in strong radiosensitzation in the four tested cell lines (Figs. 5a, S3a). Importantly, CTPI2 treatment alone was always more effective on survival of irradiated cancer cells compared to PARPi in combination with IR (Figs. 5a, S3a). Even more important, CTPI2 treatment in combination with IR reduced the SF of U87-MG cell line to the similar extend compared to IR-treatment in combination with DNA-PKi (Figs. 5a, S3a). This results hint to the contribution of the genetic background to radiosensitivity.

We further investigated whether the addition of CTPI2 was suited to further increase the sensitivity of cancer cells to IR when combined with PARPi or DNAPKi. Our data demonstrated that the triple combinations including IR, CTPI2 and PARPi or DNA-PKi further potentiated the effects of radiation in combination with PARP or even DNA-PKcs inhibitors (Figs. 5a, S3a). However, combining CTPI2 with DNA-PKi further reduced the survival fraction of irradiated cancer cells, except for the NCI-H460 being most sensitive to DNA-PKi in combination with IR (Figs. 5a, S3a).

All tested cell lines displayed increased γ-H2AX foci formation 6 h after IR when combined with CTPI2 treatment (Figs. 5b, S3b), although to a different extent. Combining CTPI2 with PARPi or DNAPKi delayed the resolution of radiation-induced γ-H2AX foci in tested cell lines compared to CTPI2-treatment alone 6 h (Fig. 5b) and 24 h after IR (Fig. S3b). Here, A549 cell line displayed an increased number of radiation-induced γH2AX foci when CTPI2 treatment was combined with DNA-PKi compared to PARPi treatment (Figs. 5b, S3a).

Taken together, our data suggested that treatment with CTPI2 enhanced radiosensitivity with inhibitors of EJ-pathways (e.g., PARP or DNA-PKcs), pointing to the metabolic disturbance of HR repair pathway mimicking the HR-ness phenotype in cancer cells.

In order to explore the ability of CTPI2-treatment to render NCI-H460 cancer cells sensitive to inhibitors of EJ-pathway upon IR in vivo, we used a well-described Chick Embryo Chorioallantoic Membrane (CAM) Model established for studying tumor growth, invasion and metastasis [25]. Here, we employed the CAM model as a proof-of-concept platform for validation of CTPI2-induced sensitivity to PARP-inhibition upon IR in vivo (Fig. 5c). Treatment of NCI-H460 cells with CTPI2 alone significantly reduced tumor volume (Fig. 5d, e). Combining CTPI2 with a single dose of 5 Gy potentiated the irradiation effect by significantly reducing the tumor volume of NCI-H460 cancer cells compared to irradiation alone (Fig. 5d, e). The triple combination of CTPI2 and IR with the PARP-inhibitor PJ34 further reduced the volume of NCI-H460 tumors compared to double treatment with irradiation and either CTPI2 or PARPi (Fig. 5d, e). These data corroborated the findings obtained from the long-term colony formation assays described above (Fig. 5a) approving the potential of CTPI2-treatment to enhance radiosensitivity with DNA repair inhibitors of EJ-pathways.

## Discussion

The genetic concept of synthetic lethality allowed to design novel therapeutic approaches to cancer [31]. Tumor cells with defective BRCA1 or BRCA2 lack the ability to perform HR efficiently [31, 32] thus sensitizing *BRCA*-mutant tumors to PARP inhibitors [33]. Furthermore, accumulation of the oncometabolite D-2-HG in the context of the gain-of-function mutation of IDH has been described to mimic the HR-ness phenotype [16]. Here we propose an innovative strategy for metabolic induction of HR-ness phenotype by pharmacologic inhibition of SLC25A1 and thereby enhancing the lethality of IR alone or in multimodal combination with PARP or DNA-PKcs inhibitors. Our data demonstrated the potential of CTPI2-treatment to enhance radiosensitivity with DNA repair inhibitors of EJ-pathways in vitro and in vivo by the induced accumulation of the oncometabolite 2-HG.

Prior work from our group and from others already suggested that pharmacologic inhibition of SLC25A1 by BTA induces accumulation of the D-enantiomer of 2-HG [6, 34, 35]. In the present study we further validated the induction of D-2-HG by SLC25A1 inhibition in additional cancer cell lines by applying genetic inhibition using RNAi technology and a novel, 3rd generation small molecule inhibitor of SLC25A1, CTPI2 [17]. Mechanistically, supplementation of cell permeable D-2-HG phenocopied the effects observed by CTPI2 treatment on DNA damage, repair as well as on disturbance of redox homeostasis and energy metabolism. Thus, SLC25A1 inhibition by CTPI2 exerted its effects through metabolic reprogramming leading to accumulation of D-2-HG and was associated with inhibition of KDM4 activity.

The results obtained in this study revealed a stable and time-dependent D-2-HG induction upon CTPI2-treatment for up to 48 h after treatment. Thus, SLC25A1-inhibition by CTPI2 represented a suitable strategy for the induction of D-2-HG and thereby to affect mitochondrial energy metabolism and DNA damage repair upon irradiation. Mechanistically, the effects observed upon CTPI2-induced D-2-HG accumulation correlated to a shift of the balance of the co-enzymes and energy carrier-molecules NAD and NADP to an oxidative state.

Herein, a recent study illustrated first mechanistic aspects of the link between 2-HG accumulation and DSB repair on the level of KDM4B-mediated histone 3 lysine 9 trimethylation (H3K9me3) near DNA breaks [16]. This study emphasized a pivotal role of 2-HG-mediated suppression of HR repair and provided an excellent explanation for the relationship among oncometabolites, the DDR and DSB repair [16]. However, here we demonstrated in addition, that SLC25A1 inhibition provides a therapeutic strategy for induction of the oncometabolite D-2-HG in cells without somatic mutation in IDH1/2. Of note, SLC25A1 inhibition enhanced vulnerability to DNA repair inhibitors of EJ-pathways in irradiated lung and glioblastoma cell line models, without IDH1/2 mutations.

By using isogenic MEF cell line models with defects in NHEJ or HR pathways, we were able to prove the radiosensitizing effect of SLC25A1 inhibition in MEF cell line proficient for HR repair pathway. Furthermore, CTPI2 treatment led to increased accumulation and delay of radiation-induced Rad51 foci indicative for a metabolically induced HR-defect upon SLC25A1-inhibition, presumably involving D-2-HG accumulation. Finally, SLC25A1 inhibition rendered NCI-H460 cells sensitive to PARP-inhibition in vivo, providing a pharmacologic strategy for induction of a HR-ness phenotype by metabolic reprogramming.

Interestingly, a recent study demonstrated an additional vulnerability of IDH1/2 mutant gliomas with increased abundance of the oncometabolite D-2-HG, namely, sensitivity to histone deacetylases (HDACs) inhibitors [36]. These novel findings point to effects of D-2-HG accumulation on several distinct histone modulating processes.

The observed effects upon SLC25A1 inhibition on DNA repair corroborated earlier observations on SLC25A1-regulated structural modification of chromatin [37]. This observation highlights the role of the citrate carrier in genetic modification rather than its action as a single metabolite transporter [37]. Of note, D-2-HG accumulation induced by CTPI2-treatment did not result in pronounced DNA damage without IR but interfered with the repair of radiation-induced DNA damage, highlighting the importance of SLC25A1 for the regulation of DNA repair pathways. In order to monitor the suggested direct effects of CTPI2-induced accumulation of D-2-HG on radiation-induced DNA damage, we utilized octyl-D-2-HG supplementation. The obtained data again demonstrated that neither CTPI2 nor octyl-D-2-HG treatment-induced DNA damage without IR. However, combining IR-treatment with CTPI2 or octyl-D-2-HG potentiated the DNA damaging effects of irradiation in NCI-H460 cells, compared to IR alone. However, CTPI2 treatment in combination with IR induced DNA damage and reduced the survival of irradiated NCI-H460 cell to a higher extent compared to the octyl-D-2-HG treatment. Thus, the effects of CTPI2 treatment went beyond the effects of octyl-D-2-HG supplementation.

Here, mechanistic studies applying metabolomics are necessary to decipher the underlying metabolic reprogramming induced by SLC25A1 inhibition or by supplementation of its surrogate oncometabolite octyl-D-2-HG under the context of an active SLC25A1 transporter.

Our short-term analysis of cellular parameters revealed that octyl-D-2-HG treatment exerted comparable effects on cell proliferation, ROS, apoptosis, and cell death levels of NCI-H460 cancer cells, suggesting that this oncometabolite is responsible for the respective short effects observed upon SLC25A1-inhibition by CTPI2. The mechanisms of IR-induced cell death depend on many factors including the cell line, radiation dosage, and the state of the cell [38]. Here, our observation of CTPI2-induced apoptosis and cell death was in good accordance with the recent discovery in colorectal cancer, which indicated that knockdown of SLC25A1 significantly inhibited colorectal tumor growth by inducing apoptosis both in vitro and in vivo [39]. These results further corroborated the lethal effect of SLC25A1-inhibition on cell function and DDR, resulting in suppression of cell reproduction. Altogether these findings support a crucial role of SLC25A1 for cancer cell proliferation and survival. Not surprisingly, octyl-D-2-HG-treatment, a major metabolite induced by SLC25A1 inhibition, was recapitulating the effects of CTPI2-treatment on cell proliferation.

Furthermore, in our study, both CTPI2 and octyl-D-2-HG-treatments suppressed mitochondrial function significantly compared with non-treated control. Metabolic reprogramming is accepted as an inseparable hallmark of cancer [10] and is contributing to radioresistance of cancer cells [6, 19, 20, 40]. A clinical study has linked SLC25A1 mutation to mitochondrial complex V deficiency, implying the involvement of the citrate carrier SLC25A1 in the regulation of mitochondrial activity [41]. Our previous work demonstrated that SLC25A1 inhibition by using BTA disturbed mitochondria function, in terms of basal respiration, mitochondrial ATP production, and spare respiratory capacity [6]. Mitochondrial dysfunction observed here was in line with the previous observation that alteration of mitochondrial function was related to ROS overproduction, genomic instability, and apoptosis regulation [42]. Others linked mitochondrial dysfunction to the inhibition of tumor progression through disturbing the autophagic machinery, which interacted with NAD^+^ metabolism through AMPK and mTOR signaling pathways [43].

Accumulating evidences suggest that NAD (including NAD^+^ and NADH) and NADP (including NADP^+^ and NADPH) are the active elements which take part in various biological processes, including energy metabolism, mitochondrial function, homeostasis of cellular oxidative state, and others [44]. Our analysis of mitochondrial function and subsequent identified mitochondrial dysfunction induced by CTPI2- or octyl-D-2-HG-treatment broadly supported the observed NAD^+^/NADH and NADP^+^/NADPH disbalance, thus linking it to mitochondrial dysfunction. The phenomenon of mitochondrial dysfunction and accompanied disbalance of NAD and NADP-levels was highly likely contribute to the lethal effects of CTPI2 or octyl-D-2-HG treatment in cancer cells. Furthermore, NAD is an indispensable molecule for energy metabolism and PARP-related DNA repair process [45] and both processes are important for recovery from radiation-induced damage.

Thus, PARP-related EJ-repair pathways become important in cells with HR repair defects, e.g., in cells with loss of BRCA1 or BRCA2 function [46]. However, only the minority of cancers have BRCA1 or BRCA2 mutations that would confer sensitivity to PARP inhibitors (PARPi) [47]. Herein, somatic IDH1/2 mutations have been reported to cause a defect in HR repair through the accumulation of the oncometabolite 2-HG with associated sensitivity to PARP inhibitors [7]. In addition, other enzymes of EJ-dependent pathways, e.g., the catalytic subunit of DNA-dependent protein kinase (DNA-PKcs), might represent suitable targets in tumors with HR repair defects [48, 49].

Interestingly, emerging evidence indicates that defects in HR repair and sensitivity to PARPi can also be induced in BRCA-proficient cancers, e.g., by treatment with DNA methyltransferase inhibitors (DNMTi) [47]. In the present study, we hypothesized that SLC25A1 inhibition creates a HR-ness phenotype leading to potential synergistic effects for radiosensitization with EJ-pathways inhibitors (PARPi or DNAPKi). In fact, the combination of CTPI2 with either PARPi or DNAPKi interfered with the removal of IR-induced DNA damage and radiosensitized cancer cells to IR.

In accordance with our observation on radiation-induced DNA damage, PARPi potentiated CTPI2’s effects on cell radiosensitivity in lung and glioblastoma cell lines in vitro.

In a final step, we validated the antineoplastic effects of CTPI2-treatment observed in vitro by using an in vivo CAM model. In accordance with the in vitro effects, exposure to CTPI2-treatment led to statistically smaller tumor size compared to untreated control tumors. However, HR-ness phenotype induced by SLC25A1 inhibition significantly reduced tumor growth in combination with PARPi, thus blocking the major DNA damage repairing pathways upon IR-treatment. The obtained in vivo result recapitulated the observed in vitro effects of CTPI2 treatment.

In summary, D-2-HG induction by genetic or pharmacologic inhibition of SLC25A1 or direct treatment with octyl-D-2-HG impact cellular processes which are important for the survival of irradiated cancer cells. Mechanistically, we correlated the effects observed upon pharmacologic SLC25A1 inhibition by CTPI2 to the function of KDM4 and mitochondrial dysfunction. We concluded that SLC25A1 inhibition affects the repair of IR-induced lethal DNA lesions by inducing D-2-HG accumulation and thereby sensitizes cancer cells to PARPi in combination with IR in vitro and in vivo. Thus, SLC25A1 inhibition is suited for pharmacologic disturbance of HR repair and thereby induces radiosensitivity in combination with clinically relevant DSB repair inhibitors in cancer cells. Further efforts are necessary to unravel the potential of drug-induced metabolic reprogramming for tumor radiosensitization by understanding the underlying mechanisms of CTPI2-induced metabolic reprogramming.

## Supplementary information

Supplementary material_Revised

aj-Checklist

## Data Availability

Any additional information required to reanalyze the data reported in this paper is available from the lead contact upon request.

## Code availability

This study did not use any unpublished custom code, software, or algorithm.
