# Supplementary material for: Accumulation of oncometabolite D-2-Hydroxyglutarate by SLC25A1 inhibition: A metabolic strategy for induction of HR-ness and radiosensitivity
Source: Cell Death Dis. 2022 Jul 22;13(7):641. doi: 10.1038/s41419-022-05098-9 (PMC9307853; doi:10.1038/s41419-022-05098-9)
Supplement: Supplementary file 1 — Supplementary material_Revised [file 41419_2022_5098_MOESM1_ESM.docx]

Supplementary Material

**Accumulation of oncometabolite D-2-Hydroxyglutarate by SLC25A1 inhibition: a metabolic strategy for induction of HR-ness and radiosensitivity.**

Kexu Xiang^1^, Christian Kalthoff^1^, Corinna Münch^1^, Verena Jendrossek^1^, Johann Matschke^1#^

^1^ Institute of Cell Biology (Cancer Research), University Hospital Essen, University of Duisburg-Essen, 45147 Essen, Germany

# Correspondence: [Johann.Matschke@uk-essen.de](mailto:Johann.Matschke@uk-essen.de) (J.M.), Institute of Cell Biology (Cancer Research), University Hospital Essen, University of Duisburg-Essen, Virchowstrasse 173, 45147 Essen, Germany; Phone: +49-201-7234234; Fax: +49-201-7235904; E-mail: johann.matschke@uk-essen.de.

## Supplementary Figures


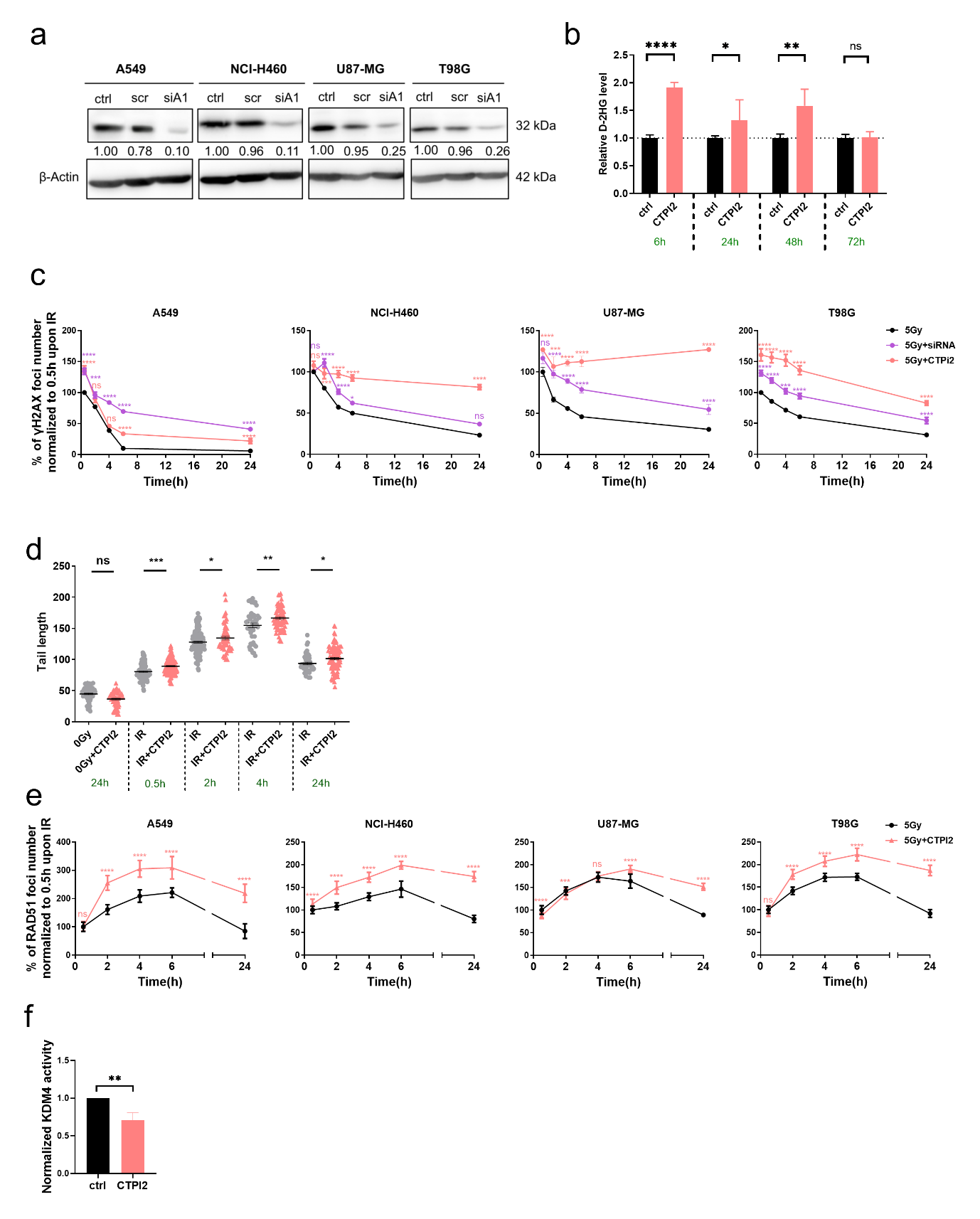


**Figure S1: Induction of D-2HG by CTPI2 and its relevance for radiation-induced DNA damage**

A549, NCI-H460, U87-MG and T98G cells were pre-treated for 2 h with CTPI2 (200 μM), without or with additional irradiation with a single dose of 30Gy, or left untreated as indicated. To achieve downregulation of SLC25A1 on protein level, cells were transfected with 45 nm siRNA pools targeting SLC25A1 or non-targeting scrambled controls (scr) with 3 μl TransIT-siQUEST and 100 μl optiMEM for 24 h in 1 ml total reaction volume, followed by medium exchange according to manufacturer's protocols.**a)** Western blots indicate SLC25A1 protein downregulation 24 h after start of transfection at the timepoint of D-2HG quantification or IR. Relative quantification is depicted with numbers under the appropriate protein band. **b)** D-2HG production of 6-72 h after CTPI2 (200 μM) treatment alone in NCI-H460 cell line was investigated using the D-2HG assay kit. **c)** Time dependent γ-H2AX foci number was counted upon SLC25A1 siRNA or CTPI2 in combination with IR (5Gy) in A549, NCI-H460, U87-MG and T98G cell lines. **d)** Time dependent (0.5-24 h) DNA damage induced by SLC25A1 inhibition (CTPI2) with IR (30Gy) as determined by alkaline comet assay in NCI-H460 cell line. **e)** Time dependent RAD51 foci number normalized to 0.5 h upon IR upon combinatory treatment of IR (5Gy) and CTPI2 or non- treatment in A549, NCI-H460, U87-MG and T98G cell lines. **f)** KDM4 activity was measured 24 h after CTPI2- or non-treatment in NCI-H460 cell line. Data represent the mean values (±SD) from three independent experiments (N=3). Statistical significance: by non-parametric unpaired t-test. ns=not significant (p > 0.05), * p < 0.05, ** p < 0.01, *** p < 0.001, **** p < 0.0001.


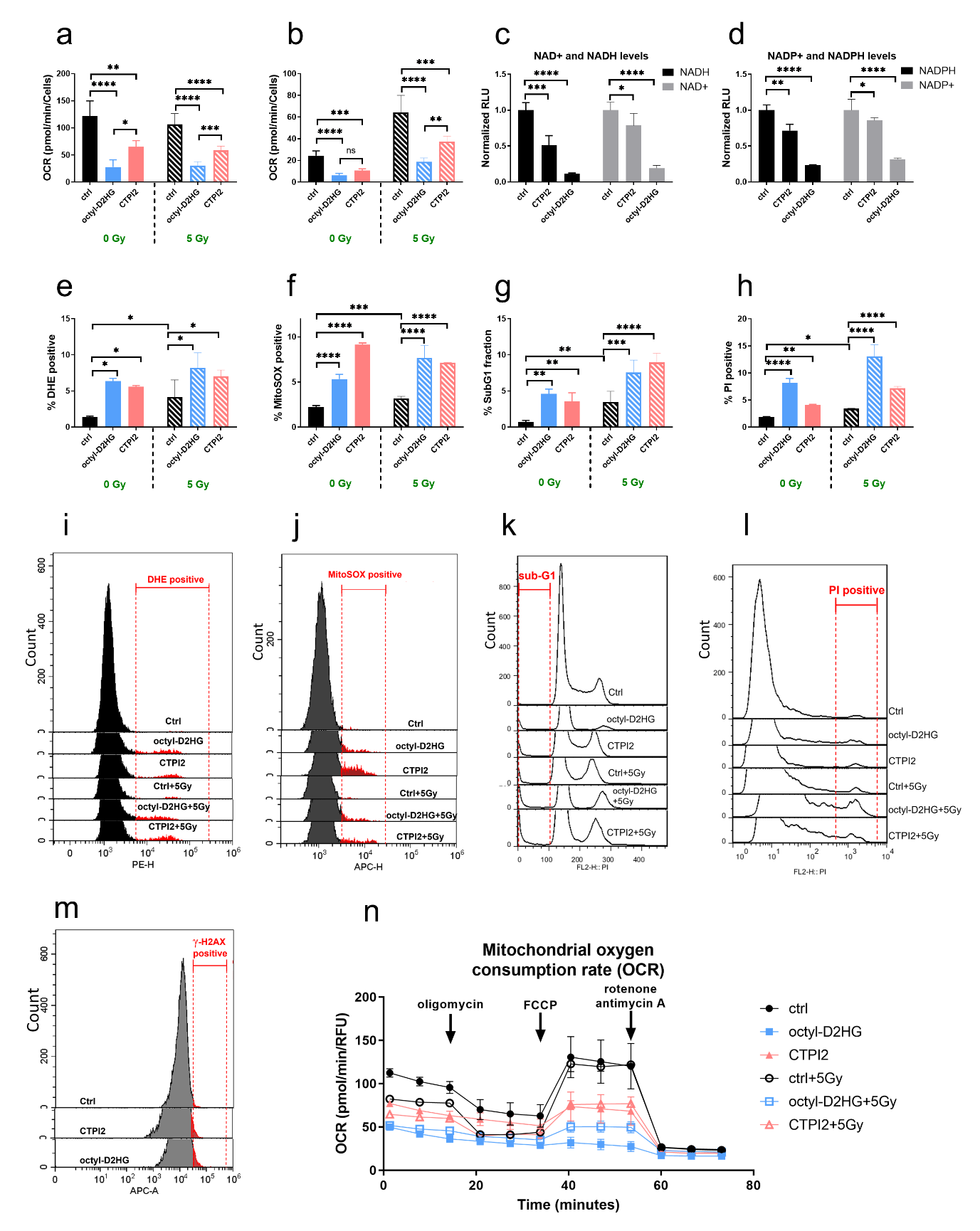


**Figure S2: Mitochondrial and cellular function upon CTPI2 or octyl-D2HG treatment with or without IR**

NCI-H460 cells were treated with CTPI2 (200 μM), octyl-D-2HG (150 μM) or solvent control without 0Gy or with single irradiation with 5Gy **a,b)** Mitochondrial function was measured 24 h after treatment by Seahorse XF96 Extracellular Flux analyser with the utilization of mitochondrial stress test. Mitochondrial function, including maximal respiration **(a)** and ATP production **(b)**, were measured 24 h after CTPI2 or octyl-D2HG treatment, with or without IR. **c, d)** Relative amounts of NAD+, NADH **(c)**, NADP and NADPH **(d)** levels in NCI-H460 cells 24 h after respective treatment. **e, f)** NCI-H460 cells were stained 6 h after treatment with DHE **(e)** or MitoSOX **(f)** to detect cytoplasmic **(e)** or mitochondrial **(f)** ROS by flow cytometry. **g)** Apoptosis was determined 48 h after treatment by staining the cells with propidium iodide (PI) in a hypotonic citrate buffer and subsequent analysis of the Sub-G1 fraction by using flow cytometry. **h)** Cell death levels were investigated by flow cytometry quantifying the % of PI-positive cells 48 h after treatment. **i, j)** Representative flow cytometry histograms of DHE **(i)** or MitoSOX **(j)** staining 6 h after indicated treatment in NCI-H460 cells. **k, l)** Representative flow cytometry histograms depicting measured apoptosis (cellular SubG1 fraction) **(k)** or cell death (%PI-positive cells) levels **(l)** 48 h after indicated treatment in NCI-H460 cells. **m)** Representative flow cytometry histogram depicting meassured γ-H2AX signal 6 h after indicated treatment in NCI-H460 cells. **n)** Oxygen consumption rate (OCR) was measured 24 h after treatment using Mito Stress Test Kit and normalized to Hoechst 33342 fluorescence units (RFU). Real-time Injection of Oligomycin (Oligo, 1 μM), FCCP (2 μM), Rotenone (Rot, 0.5 μM) and Antimycin A (AA, 0.5 μM) was performed, and OCR was measured. Data represent the mean values (±SD) from three independent experiments (N=3). one way ANOVA followed by Bonferroni post-test. * p < 0.05, ** p < 0.01, *** p < 0.001, **** p < 0.0001.


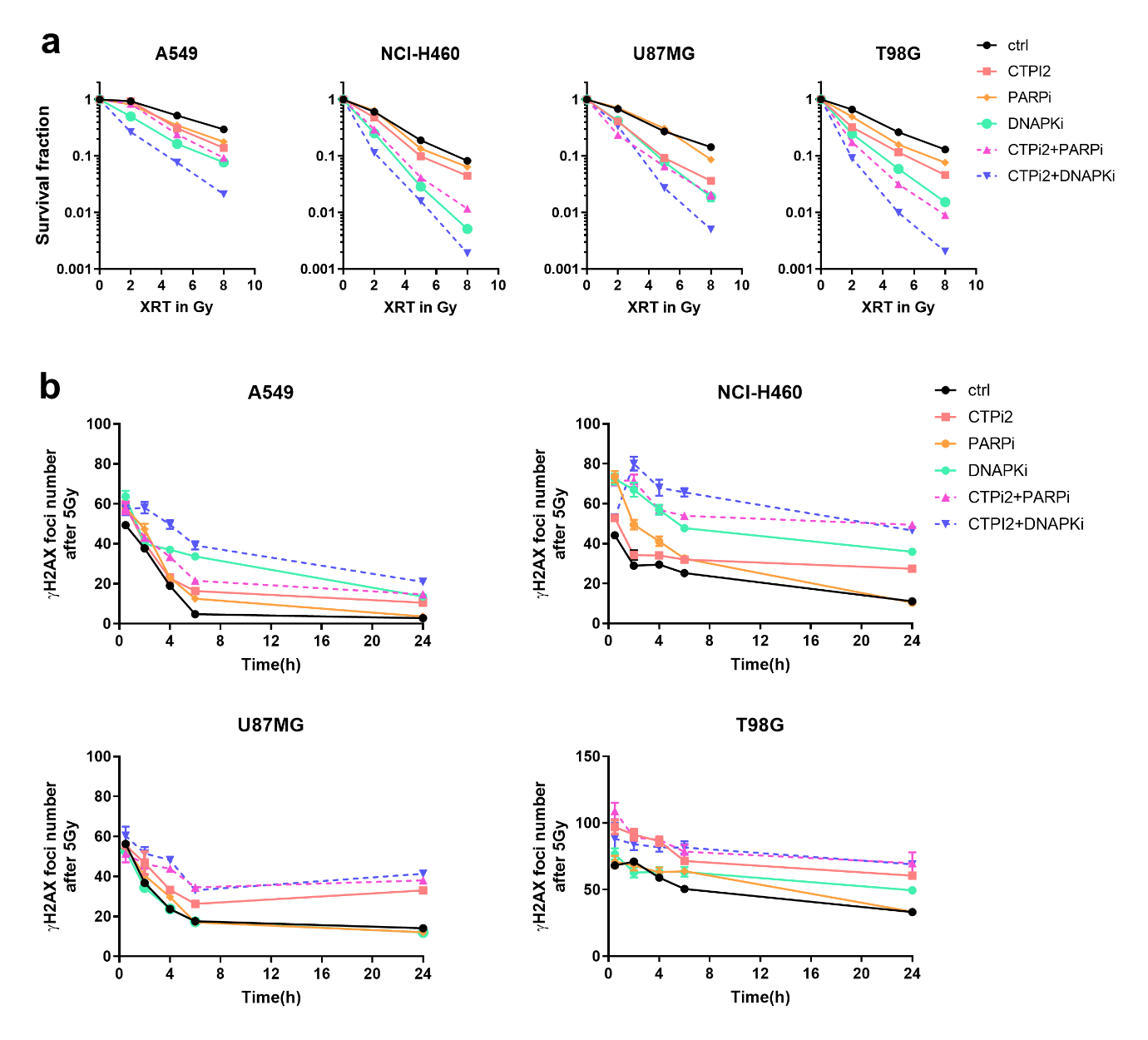


**Figure S3: SLC25A1 inhibition combined with end-joining (EJ) inhibitors on DNA damage and cell long-term survival**

A549, NCI-H460, U87-MG and T98G cells were pre-treated for 2 h with CTPI2 (200 μM), PARPi (PJ34 at 4 µM), DNAPKi (NU7447 at 4 µM) or their combinations as indicated and irradiated with a dose of 5Gy. **a)** Survival fraction of colony formation assay was calculated 8 days after treatment of CTPI2 (200 μM), PARPi (PJ34 at 4 µM), DNAPKi (NU7447 at 4 µM) or their combinations in these 4 cell lines with different doses of IR (0Gy, 2Gy, 5Gy or 8Gy). **b)** Number of γ-H2AX foci was counted at 0.5 h, 2 h, 4 h, 6 h and 24 h after combinatory treatment of IR (5Gy) and CTPI2 (200 μM), PARPi (PJ34 at 4 µM), DNAPKi (NU7447 at 4 µM) or their combinations in these 4 cell lines. Data represent the mean values (±SEM) from three independent experiments (N=3).
